# Interaction of sex and diabetes on the association between hemoglobin glycation index, hemoglobin A1c and serum uric acid

**DOI:** 10.1186/s13098-022-00955-1

**Published:** 2022-12-04

**Authors:** Ying Wei, Zhenyu Wu, Ying Wang, Guang Wang, Jia Liu

**Affiliations:** 1 Department of Endocrinology, Beijing Chao-Yang Hospital, Capital Medical University, No. 8, Gongti South Road, Chaoyang District, Beijing, China; 2 Health Management Center, Beijing Chao-Yang Hospital, Capital Medical University, Beijing, China

**Keywords:** Hemoglobin glycation index, Glycated hemoglobin A, Uric acid, Sex, Diabetes mellitus

## Abstract

**Background:**

Hemoglobin glycation index (HGI), which is calculated by blood glucose and hemoglobin A1c(HbA1c), reflects the individual discrepancy in HbA1c. This study aimed to investigate the association between HGI/HbA1c and serum uric acid(SUA) stratified by sex and diabetes.

**Methods:**

The study recruited 33772 participants who underwent physical examinations between April 2016 and August 2021 in Beijing Chao-Yang Hospital. A random subsample of 3000 subjects was utilized to calculate the formula of HGI and data of the remaining 30772 participants were used for analysis. HGI and HbA1c were categorized according to quartiles (Q1, Q2, Q3, Q4), using Q1 as the reference. We used multiple linear regression and restricted cubic splines for data analysis.

**Results:**

30772 participants with a mean age of 44.4 years old were included in the analysis, 48.6% (N = 14944) of which were female and 7.7% (N = 2363) with diabetes. Associations of HGI, HbA1c and SUA were modified by sex and diabetes. The relationship between SUA levels and HGI was positive in women without diabetes, with one unit increase in HGI associating with an 11.3 μmol/L increase in SUA (*P* < 0.001) after adjusting for other confounders. On average, each one-unit increase in HbA1c was associated with a 14.3 μmol/L decrease in SUA in women with diabetes, a 14.9 μmol/L decrease in SUA in men with diabetes, and a 16.5 μmol/L increase in SUA in women without diabetes (all *P* < 0.001). The SUA levels in men without diabetes showed a bell-shaped relation with HbA1c, increasing as the HbA1c rose to around 5.7% and then falling with a further increase of HbA1c (*P* < 0.001).

**Conclusions:**

SUA levels were inversely correlated with HbA1c in diabetic patients, also in men with prediabetes (HbA1c ≥ 5.7%), but positively correlated with HbA1c and HGI in women without diabetes. Glycemic control may help to reduce the risk of hyperuricemia in non-diabetes women.

**Supplementary Information:**

The online version contains supplementary material available at 10.1186/s13098-022-00955-1.

## Background

Hyperuricemia or high serum uric acid (SUA) levels have been shown to be associated with a series of cardiovascular and metabolic disorders, including obesity, Type 2 diabetes mellitus (T2DM), hypertension, and diabetic kidney disease [1–4]. It can not only increase the risk of gout, but also is a concomitant disease of metabolic syndrome, which contains abdominal obesity, glucose intolerance, dyslipidemia, and hypertension. Previous studies have reported that the mechanism between elevated SUA and development of metabolic diseases and vascular diseases may associate with insulin resistance [5], intrarenal hemodynamic dysfunction [6], inflammation [7], and oxidative stress [8]. Hemoglobin A1c (HbA1c), a marker of glycemic control within the prior two to three months, has become a diagnosis index of diabetes [9]. Although there have been some studies indicating that high SUA levels are associated with an increased risk of incident T2DM [4], especially in women [10, 11], research exploring SUA levels and glycemic control (HbA1c) was limited, and reported contradictory results. One study of 23,933 subjects coming from physical examinations indicated that increased SUA levels lowered the risk of elevated HbA1c(> 6.5%) [12]. Another study showed that SUA was inversely correlated with HbA1c in newly diagnosed T2DM with hyperinsulinemia. However, one study enrolling 6670 participants in China reported that SUA levels were negatively associated with HbA1c in T2DM patients but positively associated with HbA1c in normal-glucose subjects [13]. Also, another study of 1636 community‑dwelling persons conducted in Japan suggested SUA levels were inversely correlated HbA1c in men, but positively correlated with HbA1c in women [14]. Furthermore, there was one study indicating the association between SUA and HbA1c in women was not linear, positive as the HbA1c rose to 7% and then become negative with the further increase of HbA1c [15]. However, none of the prior studies exploring the association between SUA and HbA1c conducted subgroup analyses by diabetes and sex altogether.

Some studies showed that individual differences in HbA1c existed since the actual measured HbA1c of some patients was not consistent with their predicted levels, compared with other individuals with similar blood glucose levels [16, 17]. A method to measure the discrepancy between actual HbA1c and the predicted HbA1c, which is derived by putting the individual’s fasting blood glucose (FBG) into a population linear regression equation of HbA1c and FBG, has been developed and is denoted hemoglobin glycation index (HGI) [18]. HGI was found to be positively associated with cardiovascular disease, microvascular and macrovascular complications, and mortality in patients with diabetes [16]. It was also reported that high HGI was related to some cardiometabolic risk factors including triglycerides, SUA, fasting insulin, inflammatory markers, carotid atherosclerosis and nonalcoholic fatty liver disease in non-diabetic individuals [19, 20].

The question arises whether the association between HGI and SUA is also modified by sex and diabetes. Given the inconsistent results of the previous studies, to gain a deeper insight into the association between glycemic control and SUA, we aim to investigate the relationship between HGI and SUA in a larger cohort in China, also explore the association between HbA1c and SUA, accounting for both the interactive effect of sex and diabetes altogether.

## Material and methods

### Study population

The study recruited all participants who underwent physical examinations between April 2016 and August 2021 in Beijing Chao-Yang Hospital. Our exclusion criteria were as follows: (1) age < 18 years old; (2) missing data in FBG, HbA1c, or SUA since they were the parameters of our interest; (3) severe renal dysfunction (estimated glomerular filtration rate (eGFR) < 30 ml/min/1.73m^2^); (4) severe liver dysfunction (denoting as alanine transaminase or aspartate transaminase exceeded three times the upper limit of the normal range); (5) severe anemia (hemoglobin < 60 g/L). Finally, we included 33772 participants in our study. A random subsample of 3000 subjects was used to calculate the linear relationship between HbA1c and FBG. Then the other 30772 participants were used for analysis. Informed consent was obtained from all participants. The study was approved by the Ethical Review Board at Beijing Chao-Yang Hospital (Approval number: 2022-517).

### Clinical parameter measurements

Medical information of all participants was regularly collected during their physical examination by qualified physicians, including sex, age, weight, height, and medical history. We calculated body mass index (BMI) by a person’s weight in kilograms divided by the square of height in meters, and categorized it into four groups: underweight (< 18.5 kg/m^2^), normal weight (18.5 ≤ BMI < 25 kg/m^2^), overweight (25 ≤ BMI < 30 kg/m^2^) and obese (BMI ≥ 30 kg/m^2^) [21]. Venous blood samples were obtained in the morning after the participants had more than 8 h overnight fasting. The samples collected were immediately stored on ice at 4 °C and processed as soon as possible. Laboratory biochemical parameters were measured in clinical laboratories in Beijing Chao-Yang Hospital using the standard laboratory procedures. These parameters included: hemoglobin (HGB), total cholesterol (TC), high-density lipoprotein cholesterol (HDL-C), low-density lipoprotein cholesterol (LDL-C), triglyceride (TG), blood urea nitrogen (BUN), creatinine, homocysteine (Hcy), uric acid (UA), FBG, and HbA1c. eGFR was calculated by the Chronic Kidney Disease Epidemiology Collaboration (CKD-EPI) Eq.  [22]: eGFR = 141 * min (Scr/κ, 1)^α^ * max(Scr/κ, 1)^−1.209^ * 0.993^Age^ * 1.018 [if female] * 1.159 [if black], where Scr is serum creatinine, κ is 0.7 for females and 0.9 for males, α is − 0.329 for females and − 0.411 for males, min indicates the minimum of Scr/κ or 1, and max indicates the maximum of Scr/κ or 1. According to the 2019 Guideline for the diagnosis and management of hyperuricemia and gout in China, SUA concentrations greater than 420 μmol/L was defined as hyperuricemia, both in female or male [23]. Dyslipidemia was defined as TC ≥ 5.2 mmol/L, or LDL-C ≥ 3.4 mmol/L, or non-HDL-C ≥ 4.1 mmol/L, or TG ≥ 1.7 mmol/L [24]. According to the American Diabetes Association guidelines, the criteria for the diagnosis of diabetes were: FBG ≥ 7.0 mmol/L or HbA1c ≥ 6.5% (48 mmol/mol) or a random plasma glucose ≥ 11.1 mmol/L with classic symptoms of hyperglycemia [9].

### HGI calculation

Data coming from a random subsample of 3000 subjects was utilized to establish the univariate linear regression between HbA1c and FBG (predicted HbA1c (%) = 3.121 + FBG (mmol/L) * 0.484). Then predicted HbA1c and HGI were calculated for the remaining 30772 participants (HGI = observe HbA1c—predicted HbA1c) [17]. We categorized HGI (%) and HbA1c (%) according to quartiles in each subgroup groups when doing subgroup analysis (Additional file 1: Table S1).

### Statistical analysis

We described continuous variables as mean ± standard deviation (SD) and median (maximums and minimums), and categorical variables as count (percentage) in our study. Previous studies show that sex and diabetes may be potential modifiers between HbA1c and SUA [13, 14]. We used likelihood-ratio test to evaluate interaction terms for SUA and conducted subgroup analysis according to sex and diabetes. We used multiple linear regression to access the association between HGI, HbA1c, and SUA after adjusting for demographic factors (age), some medical examination parameters (BMI, HGB, TC, LDL-C, TG, BUN, and Hcy), and previous medical history (hypertension, dyslipidemia). Since a previous study indicated that SUA levels are non-linearly correlated with the levels of HbA1c [15], we used restricted cubic splines [25] with internal knots at the 25th, 50th, and 75th percentiles of the scale to assess the effect of HGI and HbA1c on SUA when they're treated as continuous variables. Other continuous variables were naturally log-transformed or used by restricted cubic splines for analysis in regression if violating the linearity assumption. We performed multiple imputations using chained equations (MICE) by the R package *mice* for missing data [26]. The type 1 error (α) for rejecting the null hypothesis was set at 0.05. Analyses were carried out using RStudio version 4.1.2.

## Results

### Sample characteristics and descriptive statistics

As seen in Table 1, 30772 participants were included in the analysis, 48.6% (N = 14944) of which were female and with a mean age of 44.4 years old. 7.7% (N = 2363) of subjects were with diabetes and 19.6% (N = 6020) were with hyperuricemia. We categorized HGI (%) and HbA1c (%) according to quartiles and the scale was as follows: HGI: Q1 (− 6.810 ≤ HGI ≤ -0.235), Q2 (− 0.235 < HGI ≤ -0.004), Q3 (− 0.004 < HGI ≤ 0.231), Q4 (0.231 < HGI ≤ 5.300); HbA1c: Q1 (2.7 ≤ HbA1c < 5.2), Q2 (5.2 ≤ HbA1c < 5.5), Q3 (5.5 ≤ HbA1c < 5.7), Q4 (5.7 ≤ HbA1c ≤ 15.1). Subjects in the highest quartile of HGI had the highest percentage of overweight or obesity, diabetes, hypertension, and dyslipidemia. With the increase of HGI categories, TC, LDL-C, and HbA1c of participants increased as well, while eGFR decreased. In addition, participants in the highest quartile of HGI had the oldest age, the highest levels of TG, BUN, SUA (Q1 vs Q2 vs Q3 vs Q4: 346 ± 92 vs 342 ± 92 vs 342 ± 90 vs 349 ± 90 μmol/L), and the lowest HDL-C. As for HbA1c, there was a crude positive association between HbA1c and SUA (Q1 vs Q2 vs Q3 vs Q4: 330 ± 89.4 vs 336 ± 90.1 vs 348 ± 92.8 vs 359 ± 89.4 μmol/L), and hyperuricemia (Q1 vs Q2 vs Q3 vs Q4: 15.7% vs 17.4% vs 20.7% vs 23.1%).

**Table 1:** Baseline information of subjects categorized by HGI quartiles

Variables	Q1 (N = 7697)	Q2 (N = 7695)	Q3 (N = 7694)	Q4 (N = 7686)	Overall (N = 30772)
Clinical data					
Sex					
Male	4260 (55.3%)	3870 (50.3%)	3747 (48.7%)	3951 (51.4%)	15828 (51.4%)
Female	3437 (44.7%)	3825 (49.7%)	3947 (51.3%)	3735 (48.6%)	14944 (48.6%)
Age(years)					
Mean (SD)	42.1 (12.8)	42.1 (13.0)	43.8 (13.8)	49.6 (15.1)	44.4 (14.1)
Median [Min, Max]	41.0 [19.0, 94.0]	40.0 [18.0, 94.0]	42.0 [18.0, 94.0]	49.0 [18.0, 98.0]	43.0 [18.0, 98.0]
BMI Categories					
Underweight	209 (2.7%)	226 (2.9%)	199 (2.6%)	146 (1.9%)	780 (2.5%)
Normal weight	4480 (58.2%)	4532 (58.9%)	4495 (58.4%)	3860 (50.2%)	17367 (56.4%)
Overweight	2566 (33.3%)	2494 (32.4%)	2503 (32.5%)	2925 (38.1%)	10488 (34.1%)
Obese	442 (5.7%)	443 (5.8%)	497 (6.5%)	755 (9.8%)	2137 (6.9%)
Diabetes					
No	7208 (93.6%)	7502 (97.5%)	7426 (96.5%)	6273 (81.6%)	28409 (92.3%)
Yes	489 (6.4%)	193 (2.5%)	268 (3.5%)	1413 (18.4%)	2363 (7.7%)
Hypertension					
No	6361 (82.6%)	6518 (84.7%)	6548 (85.1%)	5967 (77.6%)	25,394 (82.5%)
Yes	1336 (17.4%)	1177 (15.3%)	1146 (14.9%)	1719 (22.4%)	5378 (17.5%)
Hyperuricemia					
No	6111 (79.4%)	6222 (80.9%)	6263 (81.4%)	6156 (80.1%)	24752 (80.4%)
Yes	1586 (20.6%)	1473 (19.1%)	1431 (18.6%)	1530 (19.9%)	6020 (19.6%)
Dyslipidemia					
No	4115 (53.5%)	3950 (51.3%)	3839 (49.9%)	3180 (41.4%)	15084 (49.0%)
Yes	3582 (46.5%)	3745 (48.7%)	3855 (50.1%)	4506 (58.6%)	15688 (51.0%)
Laboratory Results					
HGB(g/L)					
Mean (SD)	148 (15.2)	145 (15.2)	144 (15.7)	144 (16.0)	145 (15.6)
Median [Min, Max]	148 [73.0, 200]	145 [70.0, 191]	144 [70.0, 193]	144 [66.0, 219]	145 [66.0, 219]
TC(mmol/L)					
Mean (SD)	4.79 (0.888)	4.86 (0.877)	4.90 (0.906)	4.99 (0.989)	4.88 (0.919)
Median [Min, Max]	4.71 [1.97, 11.6]	4.78 [2.24, 11.7]	4.83 [2.05, 13.9]	4.92 [2.10, 12.3]	4.81 [1.97, 13.9]
HDL-C(mmol/L)					
Mean (SD)	1.35 (0.353)	1.37 (0.360)	1.36 (0.354)	1.30 (0.347)	1.35 (0.354)
Median [Min, Max]	1.30 [0, 3.20]	1.30 [0, 3.00]	1.30 [0, 3.00]	1.22 [0.500, 3.38]	1.30 [0, 3.38]
LDL-C(mmol/L)					
Mean (SD)	2.84 (0.797)	2.91 (0.806)	2.97 (0.838)	3.08 (0.898)	2.95 (0.840)
Median [Min, Max]	2.80 [0.500, 8.50]	2.88 [0.300, 7.55]	2.90 [0.400, 9.40]	3.00 [0.500, 10.2]	2.90 [0.300, 10.2]
TG(mmol/L)					
Mean (SD)	1.52 (1.39)	1.42 (1.15)	1.44 (1.13)	1.65 (1.45)	1.51 (1.29)
Median [Min, Max]	1.15 [0.230, 33.2]	1.12 [0.170, 27.0]	1.16 [0.210, 23.2]	1.34 [0.200, 39.6]	1.19 [0.170, 39.6]
BUN(mmol/L)					
Mean (SD)	5.03 (1.26)	5.03 (1.24)	5.08 (1.27)	5.29 (1.29)	5.11 (1.27)
Median [Min, Max]	4.91 [1.57, 16.0]	4.90 [1.92, 12.9]	4.93 [1.46, 13.0]	5.15 [0.86, 16.6]	4.97 [0.86, 16.6]
eGFR(mL/min/1.73m^2^)					
Mean (SD)	109 (13.4)	108 (13.5)	107 (14.1)	103 (15.3)	107 (14.3)
Median [Min, Max]	110 [36.9, 151]	110 [30.3, 147]	108 [34.0, 148]	103 [30.1, 155]	108 [30.1, 155]
Hcy(m/L)					
Mean (SD)	14.9 (9.66)	14.7 (9.38)	14.5 (8.66)	14.8 (9.34)	14.7 (9.27)
Median [Min, Max]	13.0 [5.00, 186]	12.0 [5.00, 148]	12.0 [5.00, 179]	13.0 [5.00, 179]	13.0 [5.00, 186]
SUA(μmol/L)					
Mean (SD)	346 (91.9)	342 (91.9)	342 (90.3)	349 (89.6)	345 (91.0)
Median [Min, Max]	337 [117, 764]	332 [100, 773]	332 [118, 786]	340 [127, 761]	335 [100, 786]
FBG(mmol/L)					
Mean (SD)	5.41 (1.53)	4.99 (0.86)	4.88 (0.90)	5.19 (1.73)	5.12 (1.32)
Median [Min, Max]	5.08 [3.58, 24.8]	4.84 [3.45, 18.0]	4.72 [2.92, 17.6]	4.72 [2.50, 22.1]	4.85 [2.50, 24.8]
HbA1c(%)					
Mean (SD)	5.26 (0.68)	5.42 (0.42)	5.59 (0.44)	6.15 (1.05)	5.61 (0.77)
Median [Min, Max]	5.20 [2.70, 12.2]	5.40 [4.60, 11.7]	5.50 [4.60, 11.8]	5.80 [4.80, 15.1]	5.50 [2.70, 15.1]
Predicted HbA1c(%)					
Mean (SD)	5.74 (0.74)	5.54 (0.42)	5.48 (0.44)	5.63 (0.84)	5.60 (0.64)
Median [Min, Max]	5.58 [4.85, 15.1]	5.46 [4.79, 11.8]	5.41 [4.53, 11.6]	5.41 [4.33, 13.8]	5.47 [4.33, 15.1]

*HGI* hemoglobin glycation index, *BMI* body mass index, *HGB* hemoglobin,*TC* total cholesterol, *HDL-C* high-density lipoprotein cholesterol, *LDL-C* low-density lipoprotein cholesterol, *TG* triglyceride, *BUN* blood urea nitrogen, *eGFR* estimated glomerular filtration rate, *Hcy* homocysteine, *SUA* serum uric acid, *FBG* fasting blood glucose, *HbA1c* hemoglobin A1c

### Association between HGI categories and SUA

**Fig. 1:**
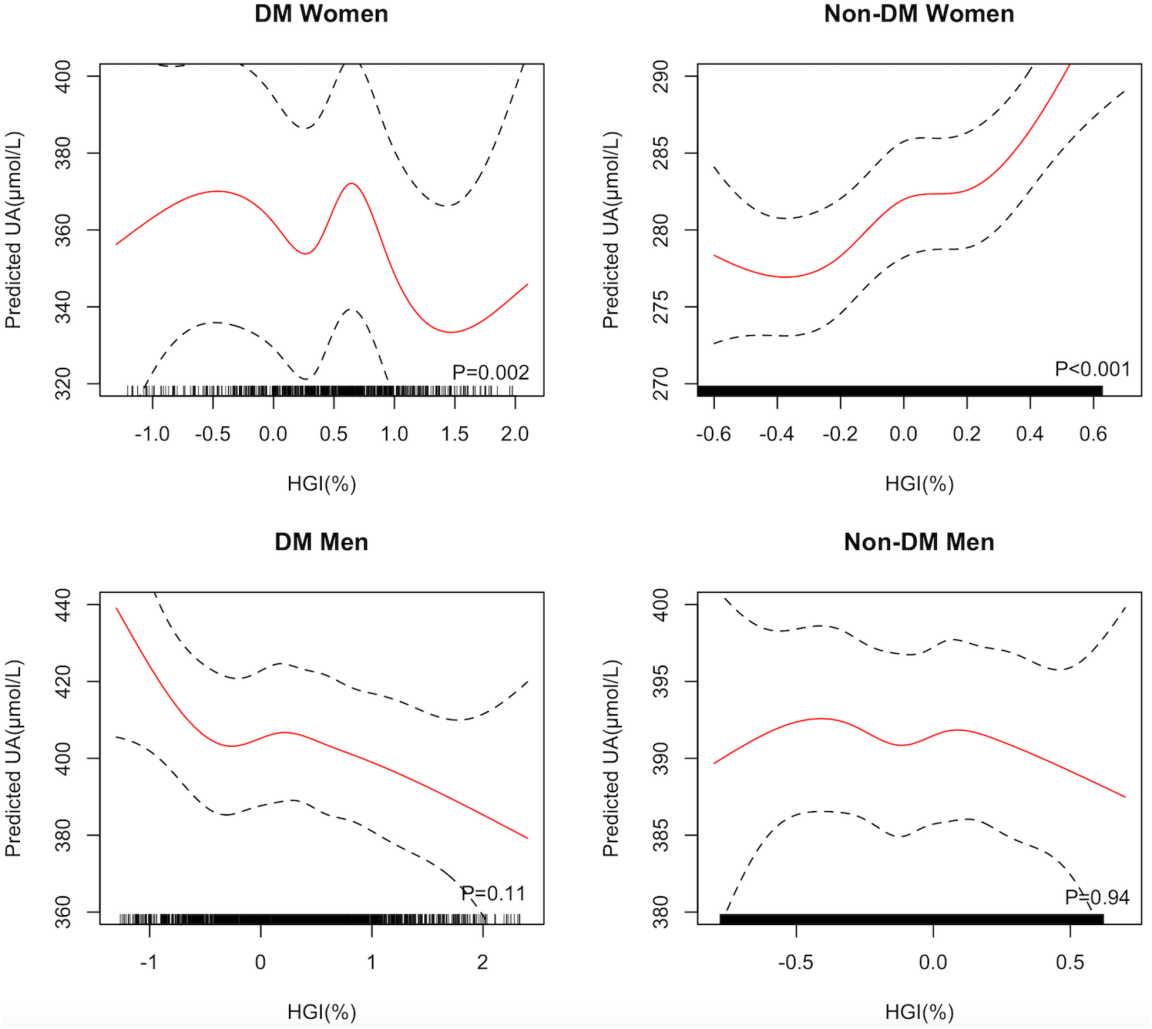
Associations between UA and HGI within sex and diabete. *HGI* hemoglobin glycation index, *UA* uric acid, *DM* diabetes mellitus. Others are the same with Table 1. Adjusting for age, BMI, HBG, TC, LDL-C, TG, BUN, Hcy, hypertension, and dyslipidemia

In the fully-adjusted models, the effects of HGI on SUA levels were different between women and men (*P* < 0.001). In addition, the effect of HGI on SUA levels differed by diabetes in women (*P* = 0.003), but the interactive effect of diabetes was not significant in men (*P* = 0.94). As shown in Table 2, in univariate analysis, the association between HGI and SUA levels was positive among females without diabetes (*P* < 0.001). After adjusting for age, HGB, BMI, TC, LDL-C, TG, BUN, Hcy, hypertension, and dyslipidemia, there was an increasing trend for SUA with the rise of HGI quartiles among women without diabetes. Women without diabetes in the highest quartile of HGI had 8.9 μmol/L higher levels of SUA (*P* < 0.001), and women without diabetes in the second quartile of HGI had 3.4 μmol/L higher levels of SUA (*P* = 0.01) compared with those in the lowest quartile. On average, each one-unit increase in HGI was associated with an 11.3 μmol/L increase in SUA (*P* < 0.001) in women without diabetes. However, as Table 2 and Fig. 1 showed, the relationship between HGI and SUA levels was non-linear in females with diabetes, and was not significant among either males with diabetes (*P* = 0.11) or males without diabetes (*P* = 0.94).

**Table 2:** Association between HGI quartiles and SUA levels

HGI	Univariate analysis		Multivariate analysis^a^	
	β(SE)	*P*	β(SE)	*P*
DM Women (N = 682)				
Q1	Ref	–	Ref	–
Q2	− 14.2(8.3)	0.10	− 12.8(7.7)	0.10
Q3	0.6(8.3)	0.49	5.3(7.7)	0.49
Q4	− 30.4(8.3)	< 0.001*	− 29.1(7.8)	< 0.001*
*P* trend	–	0.004*	–	0.006*
Non-DM women (N = 14262)				
Q1	Ref	–	Ref	–
Q2	1.9(1.5)	0.22	1.3(1.4)	0.34
Q3	6.1(1.5)	< 0.001*	3.4(1.4)	0.01*
Q4	17.2(1.5)	< 0.001*	8.9(1.4)	< 0.001*
*P* trend	–	< 0.001*	–	< 0.001*
DM men (N = 1681)				
Q1	Ref	–	Ref	–
Q2	1.5(6.0)	0.81	0.05(5.7)	0.99
Q3	0.2(6.0)	0.97	− 2.5(5.7)	0.66
Q4	− 9.8(6.0)	0.10	− 12.9(5.8)	0.03*
*P* trend	–	0.11	–	0.02*
Non-DM men (N = 14147)				
Q1	Ref	–	Ref	–
Q2	− 0.07(1.9)	0.97	− 1.4(1.8)	0.42
Q3	1.2(1.9)	0.55	0.1(1.8)	0.96
Q4	1.3(1.9)	0.51	− 2.4(1.8)	0.18
*P* trend	–	0.41	–	0.32

*HGI* hemoglobin glycation index, *SUA* serum uric acid, *DM* diabetes mellitus, *SE* standard error, *Ref* reference, others are the same with Table 1

^a^Adjusted for age, BMI, HGB, TC, LDL-C, TG, BUN, Hcy, hypertension, and dyslipidemia

^*^P < 0.05

### Association between HbA1c and SUA

**Fig. 2:**
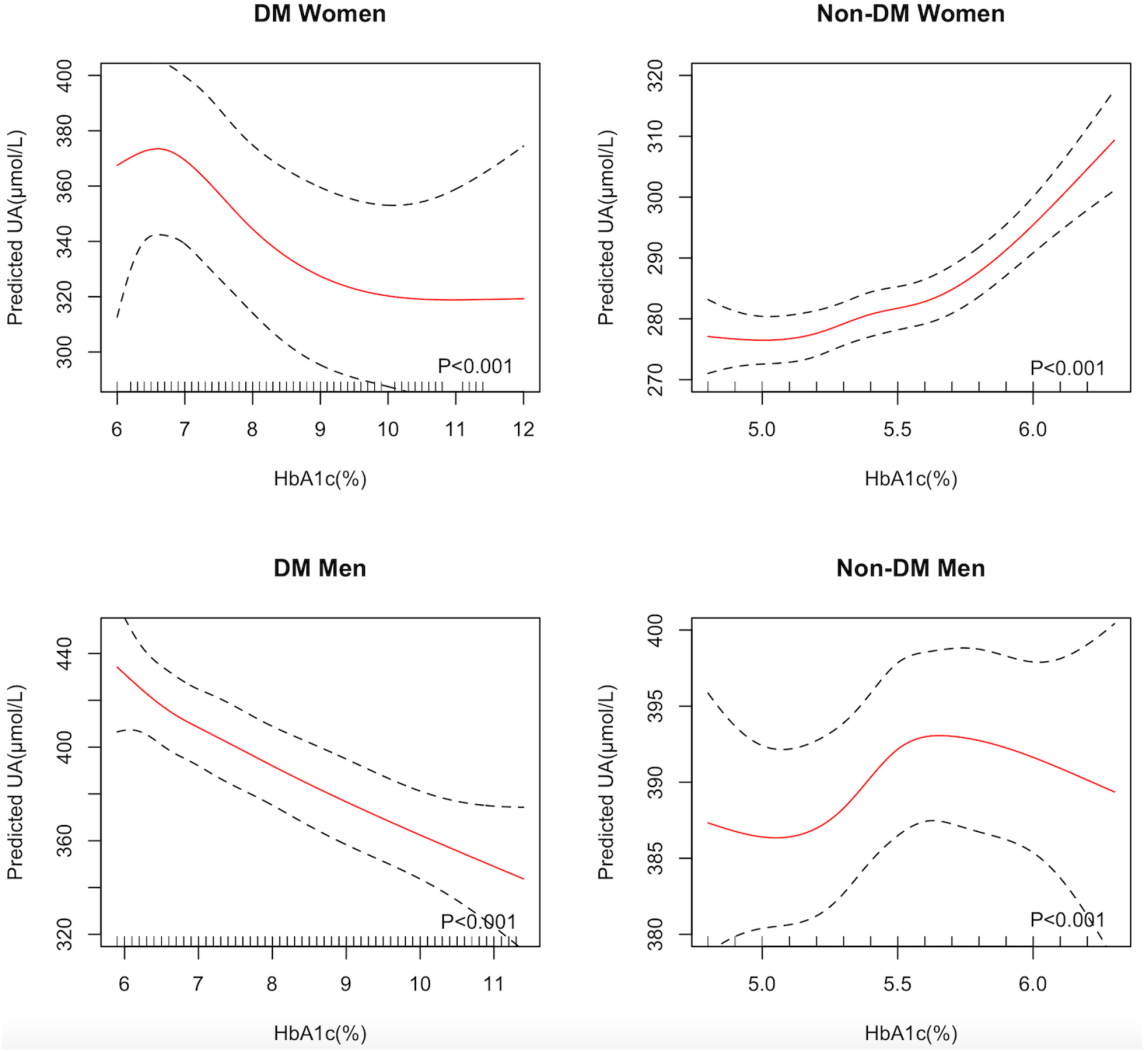
Associations between UA and HbA1c within sex and diabetes. *HbA1c* hemoglobin A1c, *UA* uric acid, *DM* diabetes mellitus. Others are the same with Table1. Adjusting for age, BMI, HBG, TC, LDL-C, TG, BUN, Hcy, hypertension, and dyslipidemia

In the fully-adjusted models, the effects of HbA1c on SUA levels were different between women and men (*P* < 0.001). In addition, the effect of HbA1c on SUA levels differed by diabetes in women (*P* = 0.02), but the interactive effect of diabetes was not significant in men (*P* = 0.53). As shown in Table 3 and Fig. 2, in women and men with diabetes, there was a declining coefficient for SUA with an increase of HbA1c quartiles, after adjusting for age, HGB, BMI, TC, LDL-C, TG, BUN, Hcy, hypertension, and dyslipidemia. Women with diabetes in the highest quartile of HbA1c had 53.0 μmol/L lower levels of SUA compared with those in the lowest quartile (*P* < 0.001). Men with diabetes in the highest quartile of HbA1c had 55.1 μmol/L lower levels of SUA compared with those in the lowest quartile (*P* < 0.001). However, in women without diabetes, there was a positive relationship between HbA1c categories and SUA. Women without diabetes in the highest quartile of HbA1c had 13.4 μmol/L higher levels of SUA compared with those in the lowest quartile (*P* < 0.001), after adjusting for all confounders in the model. On average, each one-unit increase in HbA1c was associated with a 14.3 μmol/L decrease in SUA (*P* < 0.001) in women with diabetes, a 14.9 μmol/L decrease in SUA (*P* < 0.001) in men with diabetes, and a 16.5 μmol/L increase in SUA(*P* < 0.001) in women without diabetes. The results in Fig. 2 suggested that the SUA levels in men without diabetes showed a bell-shaped relation with HbA1c, increasing as the HbA1c rose to around 5.7% and then falling with a further increase of HbA1c (*P* < 0.001).

**Table 3:** Association between HbA1c quartiles and SUA levels

HGI	Univariate analysis		Multivariate analysis^a^	
	β(SE)	*P*	β(SE)	*P*
DM Women (N = 682)				
Q1	Ref	–	Ref	–
Q2	− 5.3(8.3)	0.52	− 10.3(7.6)	0.18
Q3	− 13.0(8.3)	0.12	− 13.3(7.6)	0.08
Q4	− 42.1(8.2)	< 0.001*	− 53.0(7.7)	< 0.001*
*P* trend	–	< 0.001*	–	< 0.001*
Non-DM women (N = 14262)				
Q1	Ref	–	Ref	–
Q2	3.1(1.4)	0.03	2.4(1.3)	0.06
Q3	9.2(1.5)	< 0.001*	5.1(1.4)	< 0.001*
Q4	30.1(1.4)	< 0.001*	13.4(1.6)	< 0.001*
*P* trend	–	< 0.001*	–	< 0.001*
DM men (N = 1681)				
Q1	Ref	–	Ref	–
Q2	− 10.0(6.1)	0.10	− 12.4(5.7)	0.03*
Q3	− 22.9(6.1)	< 0.001*	− 23.3(5.7)	< 0.001*
Q4	− 52.6(6.2)	< 0.001*	− 55.1(5.9)	< 0.001*
*P* trend	–	< 0.001*	–	< 0.001*
Non-DM men (N = 14147)				
Q1	Ref	–	Ref	–
Q2	2.3(2.1)	0.27	1.0(1.9)	0.60
Q3	8.2(2.2)	< 0.001*	5.5(2.1)	0.008*
Q4	7.8(2.1)	< 0.001*	4.1(2.1)	0.051
*P* trend	–	< 0.001*	–	0.01*

*HbA1c* hemoglobin A1c, *SUA* serum uric acid, *DM* diabetes mellitus, *SE* standard error, *Ref* reference, others are the same with Table 1

^a^Adjusted for age, BMI, HGB, TC, LDL-C, TG, BUN, Hcy, hypertension, and dyslipidemia

*P < 0.05

## Discussion

To our knowledge, this study is the first to explore the relationship between HGI, HbA1c and SUA, considering the interactive role of sex and diabetes. In conclusion, we found that the associations of HGI, HbA1c and SUA were modified by sex and diabetes. After adjusting for other risk factors, SUA levels were positively associated with HbA1c and HGI in women without diabetes. However, in men without diabetes, SUA levels showed a non-linear relationship with HbA1c, increasing as HbA1c rose to around 5.7% and then falling with a further increase of HbA1c. On the contrary, in subjects with diabetes, SUA levels were inversely correlated with HbA1c.

HbA1c is a reliable indicator to monitor long-term glycemic control within the prior two to three months. Data have shown that poorer glycemic control (HbA1c ≥ 8%) elevated risks of cardiovascular disease and mortality [27], and HbA1c was also associated with metabolic syndrome criteria [28]. However, the associations between SUA and diabetes or HbA1c were inconsistent between men and women, and between different glycemic control levels [10, 13, 14, 29]. A study by *van *et al*.* indicated SUA was associated with incident prediabetes among normoglycemia women but not among normoglycemia men, and increased SUA was also associated with incident pre-diabetes among normoglycemia individuals, but not with incident T2DM among individuals with prediabetes [29]. Similarly, these sex-specific characteristics of SUA were also reported in arteriosclerotic cardiovascular risk [30], metabolic syndrome [31], and β-cell function [32]. The SUA concentration and the prevalence of hyperuricemia in men are also generally higher than in women [33]. Therefore, it is necessary to learn the association between SUA and glycometabolism within sex and separately for different glycemic levels. In our study, the associations of HbA1c and SUA were positive in women without diabetes, and negative in men (when HbA1c > 5.7%), the trend of which was consistent with one previous study [14]. UA is the end product of purine metabolism in humans. Increased SUA levels may promote the occurrence and development of diabetes by inflammation, oxidative stress, vascular endothelial injury, and inhibiting insulin pathway [34]. The risk factors for hyperuricemia include genetic mutation, intake of purine-rich and high sugar foods, alcohol, beverages and beer. These high-calorie and high-carbohydrate diets are also likely to cause blood glucose fluctuations. UA can also inhibit the trigger of insulin signaling at receptor level through an ectonucleotide pyrophosphatase / phosphodiesterase 1 (ENPP1) recruitment [35], and induce insulin resistance by NOD-like receptor family pyrin domain containing 3(NLRP3) inflammasome activation [5]. All these factors affect glucose homeostasis and potentially make a change to HbA1c and HGI. A possible mechanism of sex differences in the association may be related to genetic mutation. *Sun *et al*.* found UA-associated genes had a relationship with the risk of T2DM, glucose metabolism and insulin secretion in a Chinese population [36]. Some variants of these alleles have a sex-specific effect on SUA levels, with the minor allele for SLC2A9 having greater influence in lowering SUA in women and the minor allele of ABCG2 elevating SUA more strongly in men [37]. SLC2A9 is also identified by sequence similarity with members of the glucose transporter (Glut) family [38]. Another potential mechanism may be estrogen-related enhancement of renal urate clearance [39]. One study showed that estrogen therapy could induce insulin resistance and hyperuricemia through activation of mineralocorticoid receptor via glucocorticoid dependent pathway [40]. *May *et al*.* showed that estrogen protected pancreatic β-cells from oxidative injury and prevented diabetes through estrogen receptor-ɑ [41]. Whether estrogen is involved in sex differences of the association between HbA1c, HGI and SUA remains unclear, and requires further exploration.

In subgroup analysis according to whether participants developed diabetes or not, we found contrasting results. Association of HGI and SUA was non-linear in women with diabetes, but positive in women without diabetes. Association of HbA1c and SUA was negative in women with diabetes, but positive in women without diabetes. The opposite association may be related to increased UA excretion by kidney in the presence of hyperglycemia. In patients with diabetes, reductions in pre-glomerular resistance will facilitate the transmission of pre-glomerular perfusion pressure to the capillary network, increasing the sum of all single-nephron GFRs and resulting in glomerular hyperfiltration [42]. The hyperfiltration state and a net tubular reabsorptive defect could further promote the excretion of UA, leading to hypouricaemia and hyperuricosuria in diabetic patients, which may be one of the reasons for this inverse correlation [43]. A study in patients with diabetes and overt nephropathy also indicated that GFR improved during hyperglycemia than during euglycemia [44]. Similarly, our results showed that in men with HbA1c greater than 5.7%, which happens to be the cut point of “prediabetes” by the American Diabetes Association [9], the association between HbA1c and SUA levels was negative (Fig. 2), consistent with the theory of renal glomerular hyperfiltration in the condition of hyperglycemia. We further did a mediation analysis of eGFR in the association between HbA1c and SUA among patients with diabetes. We found that the effect of HbA1c on SUA levels was alleviated when conditioning on eGFR, and 16% of the effect could be explained by eGFR (*P* < 0.05), which supported this hypothesis. Another potential hypothesis of the negative association between SUA and HbA1c among subjects with diabetes was about insulin. Insulin stimulates uric acid reabsorption via regulating renal urate transporters, causing increasing levels of SUA [45]. In some diabetic patients with poor glycemic control and poor pancreatic beta cell function, insulin secretion is limited, improving urinary excretion of UA. Hypouricemia due to hyperuricosuria is also an indicator of renal tubular abnormality in diabetics [46]. In the absence of diabetes, *Lou *et al*.* suggested that higher SUA levels increased the risk of T2DM in women without T2DM at baseline in a large longitudinal cohort study in China with 37,296 adults [10], which was consistent with our results that the association between HbA1c and SUA was positive in non-diabetes women.

Generally, the relationship between SUA and HbA1c, and the relationship between SUA and HGI were consistent in women without diabetes, but not in women with diabetes and men. HbA1c may possess an advantage in relating to SUA levels when compared with HGI. Interindividual variation in HbA1c driven by factors other than blood glucose concentration appears in people with and without diabetes [17]. The new marker, HGI, which is calculated by FBG and HbA1c, reflects the individual discrepancy. In our study, participants with higher HGI also had higher levels of HbA1c and other metabolic parameters, including TC, LDL-C, in line with other studies reporting that high HGI individuals displayed an unfavorable cardio-metabolic risk [19, 20]. The measurement of HGI is important since it would help to avoid misinterpretation of glycemic control and to avoid inappropriate therapeutic management [16]. It may also help physicians assess patients’ risk of cardiometabolic disease, including hyperuricemia, especially for women without diabetes. Our study indicated that glycemic control should be given attention to not only by diabetes patients but also by general populations, since poor glycemic control was associated with elevated SUA levels. Accounting for the individual discrepancy of HbA1c, a calculation of HGI in women without diabetes may also help to evaluate the risk of hyperuricemia, thus allowing for earlier prevention. Potential biological mechanisms underlying the associations remain to be further investigated.

This study has some limitations. Firstly, participants of the study only came from one health center in north China. Individuals who came to the hospital to undergo physical examination were likely to be with high education levels and high incomes, limiting the generalizability for other places and other population. Secondly, our study didn’t have data about eating habits, physical activity, and medication, which may also have an effect on SUA levels and blood glucose, and can be confounding factors. Thirdly, we did not measure relevant indicators of pancreatic cells function, and whether the relationship between SUA and HbA1c/HGI is related to insulin resistance remains to be explored. Finally, this retrospective and cross-sectional study didn’t indicate a causal association between SUA and HbA1c/HGI. But it provided new insights and laid a foundation for future biological studies.

## Conclusions

In conclusion, our study extended the observations of other studies, indicating that the associations between HbA1c/HGI and SUA levels differed by sex and diabetes. After adjusting for other risk factors, SUA levels were inversely correlated with HbA1c in diabetic patients, and also in men with prediabetes (HbA1c ≥ 5.7%). SUA levels were positively associated with HGI and HbA1c in women without diabetes. On average, each one-unit increase in HGI was associated with an 11.3 μmol/L increase in SUA, and each one-unit increase in HbA1c was associated with a 16.5 μmol/L increase in SUA in women without diabetes. To sum up, not only diabetes patients but also general populations should pay attention to glycemic control. Glycemic control may help to reduce the risk of hyperuricemia, especially in women without diabetes. A calculation of HGI in them may also help to evaluate the risk when accounting for the individual discrepancy of HbA1c.

## Supplementary Information

**Additional file 1: Table S1.** HGI and HbA1c quartiles in subgroups.

## Data Availability

The data are available from the corresponding author upon reasonable request.

## Abbreviations

BMI: Body mass index
BUN: Blood urea nitrogen
DM: Diabetes mellitus
eGFR: Estimated glomerular filtration rate
FBG: Fasting blood glucose
HbA1c: Hemoglobin A1c
Hcy: Homocysteine
HDL-C: High-density lipoprotein cholesterol
HGB: Hemoglobin
HGI: Hemoglobin glycation index
LDL-C: Low-density lipoprotein cholesterol
SUA: Serum uric acid
TC: Total cholesterol
T2DM: Type 2 diabetes mellitus
TG: Triglyceride
UA: Uric acid
