# Supplementary material for: Interaction of sex and diabetes on the association between hemoglobin glycation index, hemoglobin A1c and serum uric acid
Source: Diabetol Metab Syndr. 2022 Dec 4;14:185. doi: 10.1186/s13098-022-00955-1 (PMC9719659; doi:10.1186/s13098-022-00955-1)
Supplement: Supplementary file 1 — Additional file 1: Table S1. HGI and HbA1c quartiles in subgroups. [file 13098_2022_955_MOESM1_ESM.docx]

**Supplementary Table 1. HGI and HbA1c quartiles in subgroups**

|  |  |  | **Q1** | **Q2** | **Q3** | **Q4** |
| --- | --- | --- | --- | --- | --- | --- |
| **HGI** | DM Women  N= 682 |  | [-3.239, 0.087)  N=171 | [0.087, 0.507)  N=170 | [0.507, 0.886)  N=170 | [0.886, 5.303]  N=171 |
|  | Non-DM women  (N= 14262) |  | [-2.311, -0.219)  N=3555 | [-0.219, -0.004)  N=3566 | [-0.004, 0.210)  N=3574 | [0.210, 1.351]  N=3567 |
|  | DM men  (N=1681) |  | [-6.809, -0.174)  N=420 | [-0.174, 0.379)  N=420 | [0.379, 0.852)  N=420 | [0.852, 4.857]  N=421 |
|  | Non-DM men  (N=14147) |  | [-2.633, -0.261)  N=3537 | [-0.261, -0.035)  N=3536 | [-0.035, 0.189)  N=3536 | [0.189, 1.546]  N=3538 |
| **HbA1c** | DM Women  (N= 682) |  | [5.0, 6.6)  N=171 | [6.6, 7.1)  N=168 | [7.1, 8.0)  N=169 | [8.0, 14.7]  N=174 |
|  | Non-DM women  (N= 14262) |  | [3.1, 5.2]  N=4539 | (5.2, 5.4]  N=3533 | (5.4, 5.6]  N=2855 | (5.6, 6.4]  N=3335 |
|  | DM men  (N=1681) |  | [4.6, 6.6)  N=343 | [6.6, 7.1)  N=452 | [7.1, 8.2)  N=449 | [8.2, 15.1]  N=437 |
|  | Non-DM men  (N=14147) |  | [2.7, 5.2)  N=2379 | [5.2, 5.5)  N=4665 | [5.5, 5.7)  N=3191 | [5.7, 6.4]  N=3912 |

Abbreviations: HGI: hemoglobin glycation index, HbA1c: hemoglobin A1c, DM: diabetes mellitus.
